# Research on the application of mobile phone location signal data in earthquake emergency work: A case study of Jiuzhaigou earthquake

**DOI:** 10.1371/journal.pone.0215361

**Published:** 2019-04-12

**Authors:** Xia Chaoxu, Nie Gaozhong, Fan Xiwei, Zhou Junxue, Pang Xiaoke

**Affiliations:** 1 Institute of Geology China Earthquake Administration, Beijing, China; 2 Earthquake Administration of Guangxi Zhuang Autonomous Region, Nanning, China; Pablo de Olavide University, SPAIN

## Abstract

After an earthquake, the important task of emergency rescue work is to minimize casualties, but due to the suddenness of earthquake disasters, it is difficult to obtain enough disaster information immediately, especially personnel distribution and movement information. The traditional methods of obtaining disaster data are through reports from the disaster area or field investigations by the emergency rescue team; this work lags, and its efficiency is low. This paper analyzes the feasibility of using mobile phone location signal data in earthquake emergency rescue work in several respects, such as quantity, location, change rate, and epicentral distance. The results show that mobile phone location signal data can quickly obtain the situation of personnel distribution and quantity after an earthquake, and we find the change rate, distance, etc., can determine the approximate range of the earthquake impact field. Through the data distribution in different time periods, the movement of personnel after the earthquake can be obtained. Based on several situations, we can determine the basic situation of the disaster-stricken areas in times after the earthquake, especially the personnel relevant to the situation, and these data can provide a scientific basis for emergency rescue decision making.

## Introduction

In recent years, mobile phones have become a pervasive technology with users carrying them at almost all times. From this perspective, the ubiquity of such platforms has transformed mobile phones into one of the main sensors of human behavior. With the advent of the era of big data, mobile phones have become increasingly popular; the result is that people's dependence on mobile phones has become increasingly high. Many users carry mobile phones at any time all day long. The GPS information from the mobile phone itself and the location information obtained in the process of using the mobile phone can reflect the real-time location information of the user. From this point of view, mobile phone location signal data can be seen as indicators of human activity. This ‘geographical footprint’ is similar to that collected when a cell phone call or text message is sent. Thus, social activity with GPS coordinates can be aggregated to and summarized by various spatial/political units for different analytical purposes[1–4]. Mobile phone-related data have also been widely used in different disciplines, such as research on the movement of people, the distribution of people after natural disasters, and the spread of epidemics.

Disaster management demands an immediate decision-making process, and this work requires us to conduct comprehensive analysis based on timely and accurate information data obtained after an earthquake. The primary task of earthquake emergency rescue work is to reduce casualties. Therefore, it is particularly important to accurately understand the distribution of personnel and the movement of the population after the earthquake. The existing approaches to assess population movements in the immediate aftermath of disasters, such as transport surveys and manual registration of individuals at emergency relief hubs, are often less useful: while important for record-keeping purposes, both methods are slow and may exclude those groups who are unreachable[5–8].

China is one of the countries with the most intense seismic activity in the world and the most severe earthquake disasters[9]. Earthquake emergency work is particularly important. The prominent features of an earthquake emergency are tight time limits and heavy tasks. For earthquake emergency rescue work, acquisition of seismic emergency data and extraction of disaster information form the basis for emergency decision making. The accuracy of the data directly affects the accuracy of emergency rescue work. From the data acquisition perspective, the time after an earthquake can be divided into three periods: black box period, gray box period and white box period. These periods use the amount of disaster data obtained after an earthquake as a measure[10]. The black box period refers to the period before the first outflow of actual disaster data from the disaster area after the earthquake. It is usually a few hours after the earthquake. The gray box period refers to the period from the first time actual disaster data are transmitted to the time when 80% of the disaster data are reported. This period during which the disaster data are acquired is generally a few hours to several days after the earthquake; the period after the gray box period can be called the white box period. At this time, more than 80% of the disaster data is obtained. This period lasts from one to a dozen days after the earthquake. In the black box period after the earthquake, it is possible to obtain more accurate disaster data and obtain better results.

Many researchers have studied the application of mobile phones in postdisaster emergency rescue work. For example, mobile phone traces have been used to trace population movement behaviors or the response to large-scale emergencies in a postearthquake scenario [11] [12] [13] [14]. On the other hand, many researchers incorporate both the mobile phone location data and the spatial data, i.e. [15–18], the social networks [19–22]that address the spatial nature of human mobility in emergency work such as earthquakes and disease outbreaks. An important goal of these studies is to gain an understanding of social responses, which could be useful to implement or improve policies for future emergencies.

The core of earthquake emergency rescue work is to minimize the number of casualties. Therefore, it is crucial to obtain timely data about the affected people after the earthquake, and the traditional method to obtain the situation of the affected people is based on manual registration of individuals. However, the efficiency of this work is very low. According to mobile phone location signal data, we find that due to its characteristics of real-time availability and easy access, it provides a possibility for quickly obtaining disaster data after an earthquake. In this paper, we use mobile phone location signal data in earthquakes to study the possibility of use in emergency rescue work. For example, the distribution of earthquake hazard zones, the response of personnel, the movement of personnel, etc., could be traced. When we need to make emergency rescue decisions after an earthquake, these data can provide supporting information and a scientific basis.

According to the China Seismological Network Center, a Ms7.0 earthquake occurred in Jiuzhaigou County, Sichuan Province, on August 8, 2017, at 21:19:46, and the epicenter was located at 33.14°N, 103.79°E. The earthquake occurred at the eastern end of the eastern Kunlun fault zone of the Bayankala block on the Qinghai-Tibet Plateau, which intersects with the Huya fault and the Minjiang fault. The earthquake was moderate-strong and occurred due to the slippage of the Bayankala block on the Qinghai-Tibet Plateau. The focal depth of the earthquake was approximately 20 km (http://news.ceic.ac.cn/CC20170808211947.html), and the casualties of the earthquake amounted to 25 (http://finance.china.com.cn/hz/sh/2345/20170813/7818.shtml).

This article mainly uses mobile phone location signal data to determine people’s movements and area changes during the postearthquake stage of the Jiuzhaigou earthquake. These data consist of the rate of change in the number of signals in the same area, the rate of change in the number of signals in different areas, the amount of change in signal data, and the analysis of the relationship between signal data changes and distances to the epicenter. To obtain the possible postearthquake distribution of personnel, the direction of personnel flow, and the relationship with seismic intensity, we explore the application of mobile phone location signal data to rapid response after the earthquake.

## Method

The number of mobile phone location signals is dynamically changing. The movement of people mainly affects the location of mobile phone signal data. In general, signal data represent a person. After an earthquake occurs, the number of mobile phone signals in a certain area may change, so the change rate in the number of signals can reflect the movement of people in different regions. The formula for calculating the change rate is as follows:

$$\alpha =\frac{N_{b}-N_{a}}{N_{a}}$$ (1)

where N_a_ is the signal number of the previous stage and N_b_ is the signal number of the later stage. When α<0, the number of signals in the later phase is less than that in the previous phase. Conversely, when α> is 0, the number of signals in the later phase is greater than that in the previous phase.

In this article, the mobile phone location data adopted are real-time location data provided by Getui Company (https://www.getui.com/cn/index.html). The data acquisition format is Geohash, and the precision is 6 bits. The obtained mobile phone location signal data mainly include the time and number of mobile phone signal acquisition within a certain latitude and longitude range, do not include any personal privacy information about the mobile phone user, and do not violate relevant legal norms.

We analyze the changes in the amount of data in overlap and nonoverlap areas. The overlap area mainly refers to the change in the mobile phone position signal at a certain time after the earthquake relative to the earthquake time in the same latitude and longitude range. The nonoverlap area mainly refers to the change in the mobile phone position signal at a certain time after the earthquake in different latitude and longitude ranges with respect to the earthquake time. Through the analysis of the changes in the number of mobile phone signals from overlap areas, nonoverlap areas and distances from the epicenter, the characteristics of the movement of people after the earthquake are obtained.

The specific test methods and steps are as follows: First, the obtained original mobile phone signal data are preprocessed, and the obtained Geohash format data are converted into latitude and longitude information data through the correlation algorithm, thereby obtaining the mobile phone signal distribution data within the research area. The data are determined according to the time series. The time granularity is 1 minute. Through the data preprocessing, the number of mobile phone signals at a certain time in the study area is obtained. We collect phone location data from Jiuzhaigou County; the time period of data acquisition spans from 2 hours before the earthquake to 8 hours after the earthquake (19:00 on August 8 to 5:00 on August 9), for a total of 10 hours of data. In contrast, we also collect data for the same period on the days before the earthquake. We carry out experiments and analysis from three aspects: the distribution of signal data, the change rate in signal data, and the distance between signals and the epicenter. Considering the change in the number of phone signals before and after the earthquake in the study area, the calculation of the change rate is the basis for the correlation analysis.

Second, the obtained number of mobile phone signals is grid-processed; the data in the grid are integrated and statistically analyzed. Through analyzing the trend in the number of mobile phone signals in different periods, we obtain the change trends at different times (before the earthquake, when the earthquake hit, and after the earthquake) within the study area. We distinguish the rate of change and perform separate analyses based on overlapping regions and nonoverlapping regions. The data determined from the same research area are selected from 10 minutes before the earthquake to 10 minutes after the earthquake, and the seismic intensity map of the study area is superimposed and analyzed. Therefore, we can obtain the relationship between the distribution of mobile phone signals, the trend of change, and the actual intensity. For overlap data, if the amount of mobile phone signal data before an earthquake is the same as the amount of signal data at the time of an earthquake, then the personnel in the location have not changed or moved. The other situation is nonoverlap data; the data or location before and after the earthquake are different (for example, the same location shows data before the earthquake but no data after the earthquake, or a location has no data before the earthquake, but new data appear after the earthquake). We analyze these two kinds of data separately to obtain the change or the movement of personnel in earthquake regions. In this paper, we select the data change rates for 21:09 (10 minutes before the earthquake), 21:19 (earthquake time), and 21:29 (10 minutes after the earthquake) to analyze.

Third, we take the epicentral location as the center, and we research the relationship between the change rate in mobile phone location signal data and the epicenter location before and after the earthquake, and the range that we set is within 100 km from the epicenter.

## Results

**Fig 1:**
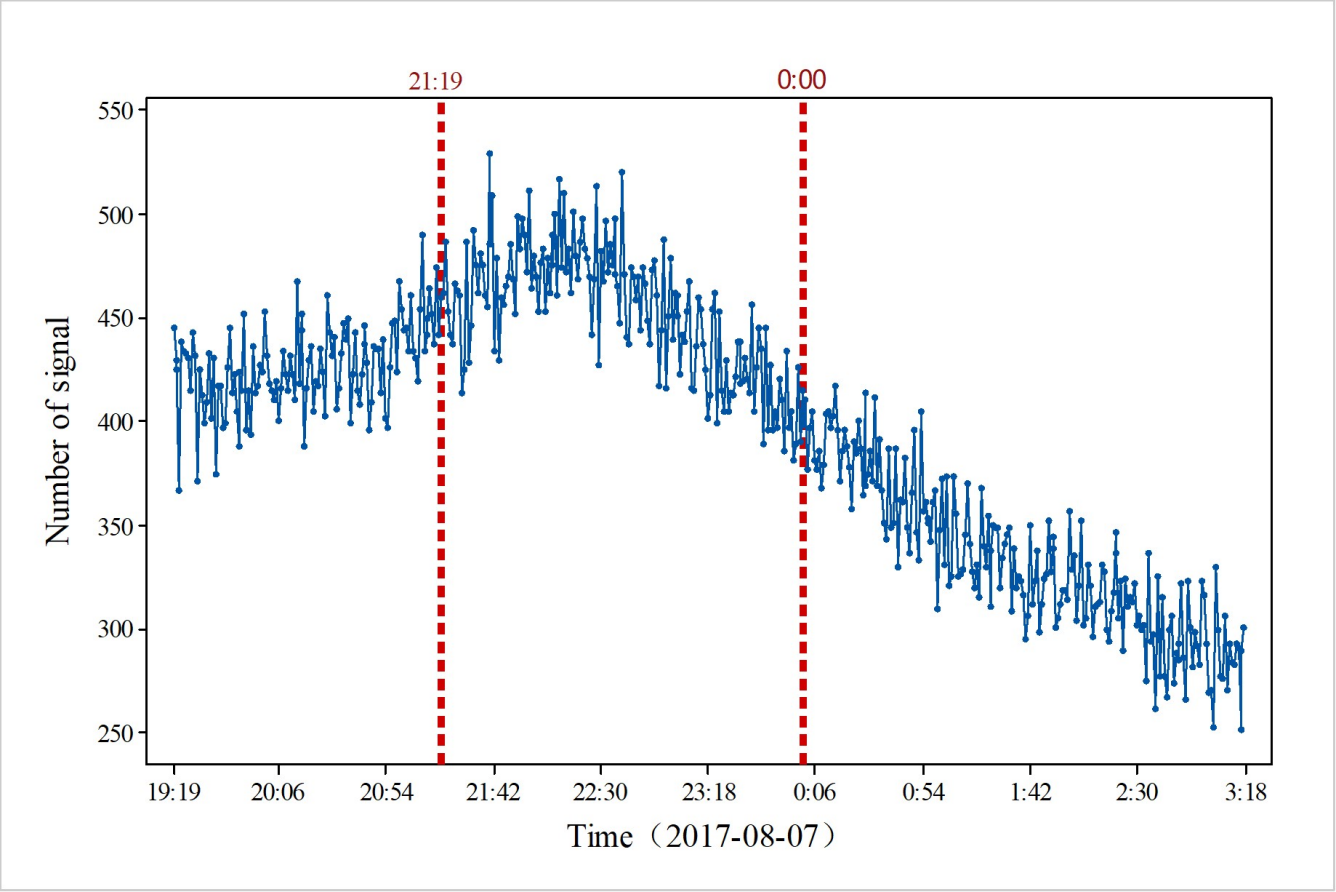
Overall changes in mobile phone location signal data in the study area on August 7, 2017.

We performed a detailed analysis of the results from three aspects. As shown in Fig 1, the change trend in the mobile phone signal data during the same time period was relatively stable on the day before the earthquake occurred (August 7, 2017). There was no significant fluctuation, especially at the time corresponding to the earthquake (21:19). The number of signals per minute was concentrated in the range of 200–500. From 19:00, the number of signals increased. The trend reached the maximum range at approximately 22:00; then, the number of signals began to decrease, showing a downward trend, but the rate of decrease was relatively stable, with no obvious transient changes.

**Fig 2:**
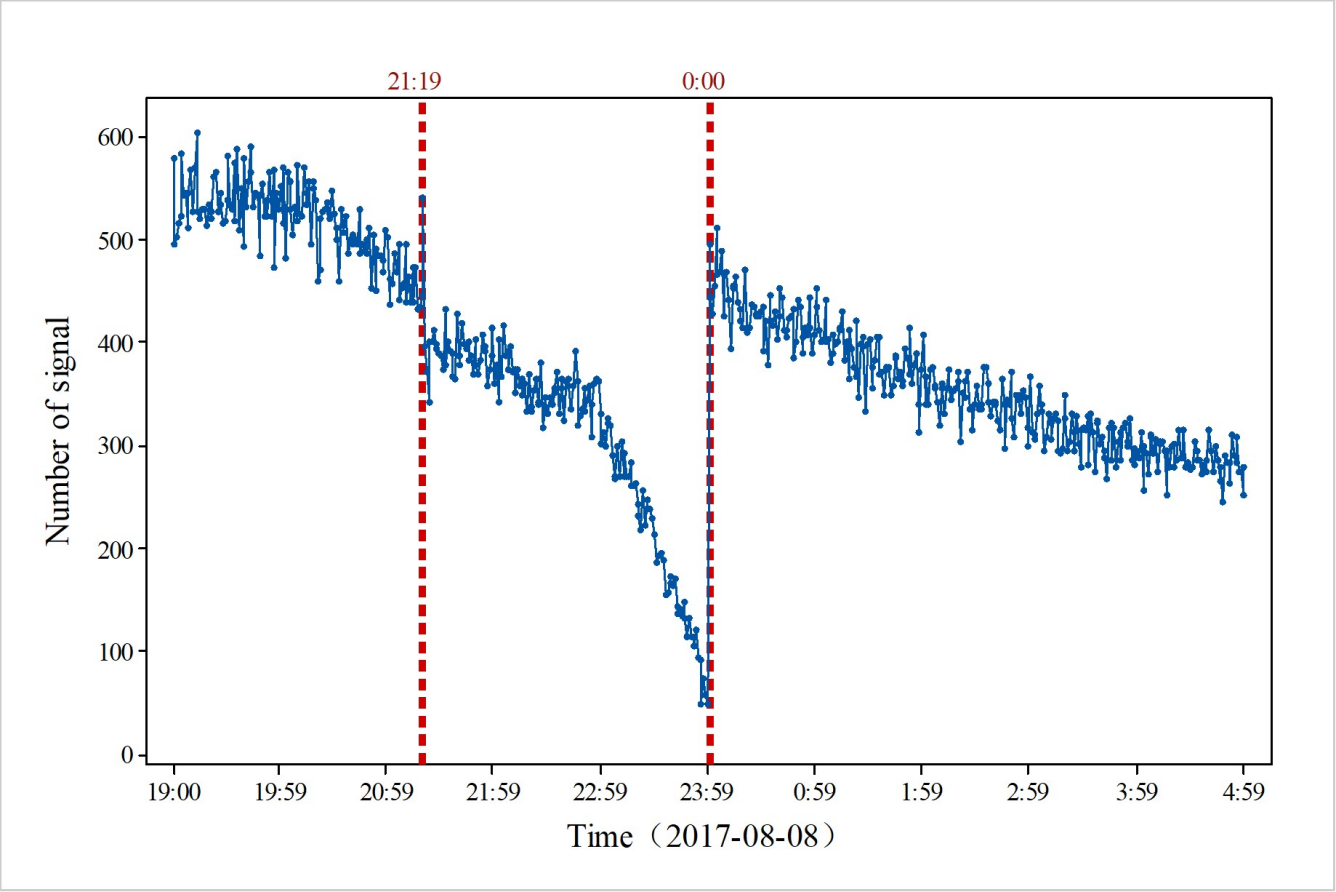
Overall changes in mobile phone location signal data in the study area on August 8, 2017.

As shown in Fig 2, since the mobile phone location signal data in the study area that we obtained were between 19:00 on August 8 and 5:00 on August 9, the data were collected according to the unit of minutes, and the data volume was large. Therefore, we obtained the location and number of mobile phone signals in the study area in each minute. According to the number of signals, the overall data show a downward trend, in which there were large amounts of change at two periods. The first stage was at 21:20. In terms of points, the amount of data decreased from 540 before the earthquake to 396 after the earthquake, with a decrease rate of 26.7%. The other phase of change is the early morning on August 9, the number of signals has increased from 100 to nearly 500.

**Fig 3:**
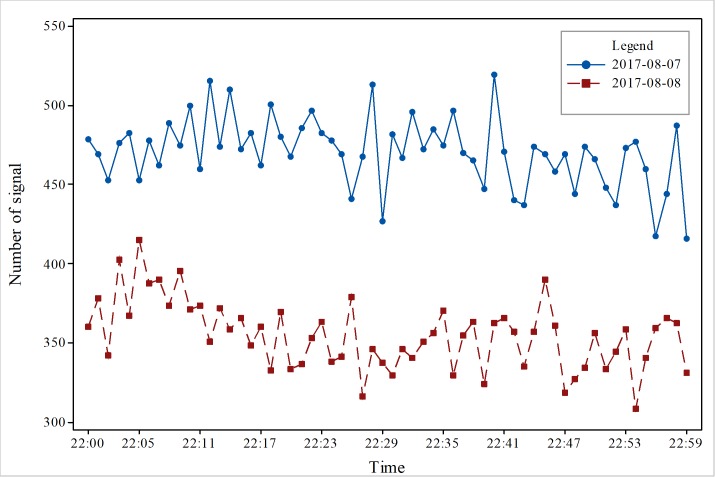
Changes in cell phone position signal data before the earthquake in the study area.

**Fig 4:**
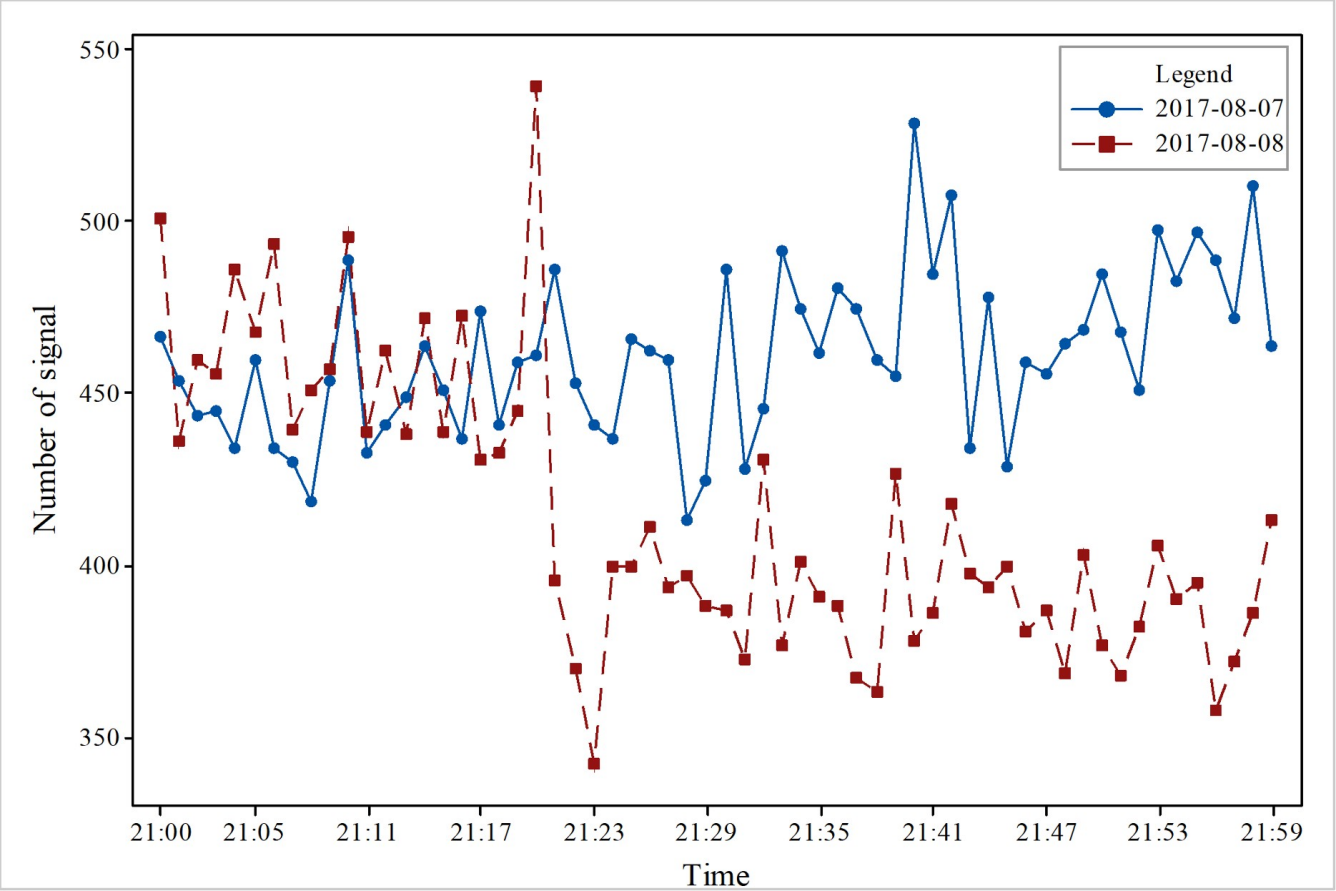
Changes in cell phone position signal data during earthquakes in the study area.

**Fig 5:**
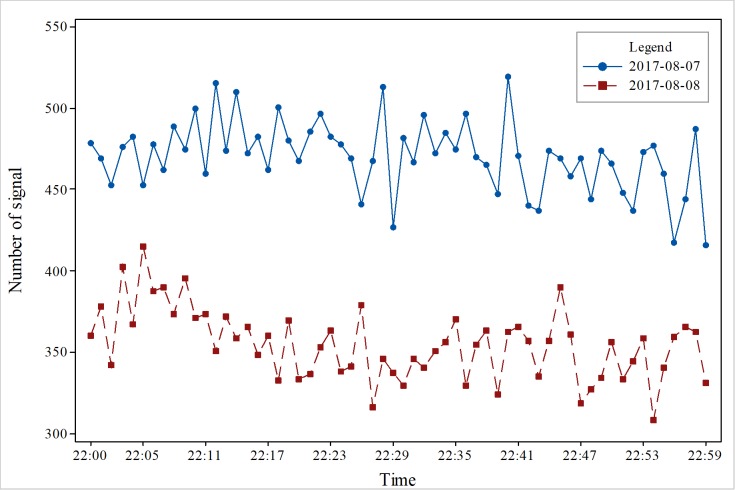
Changes in cell phone position signal data after the earthquake in the study area.

**Fig 6:**
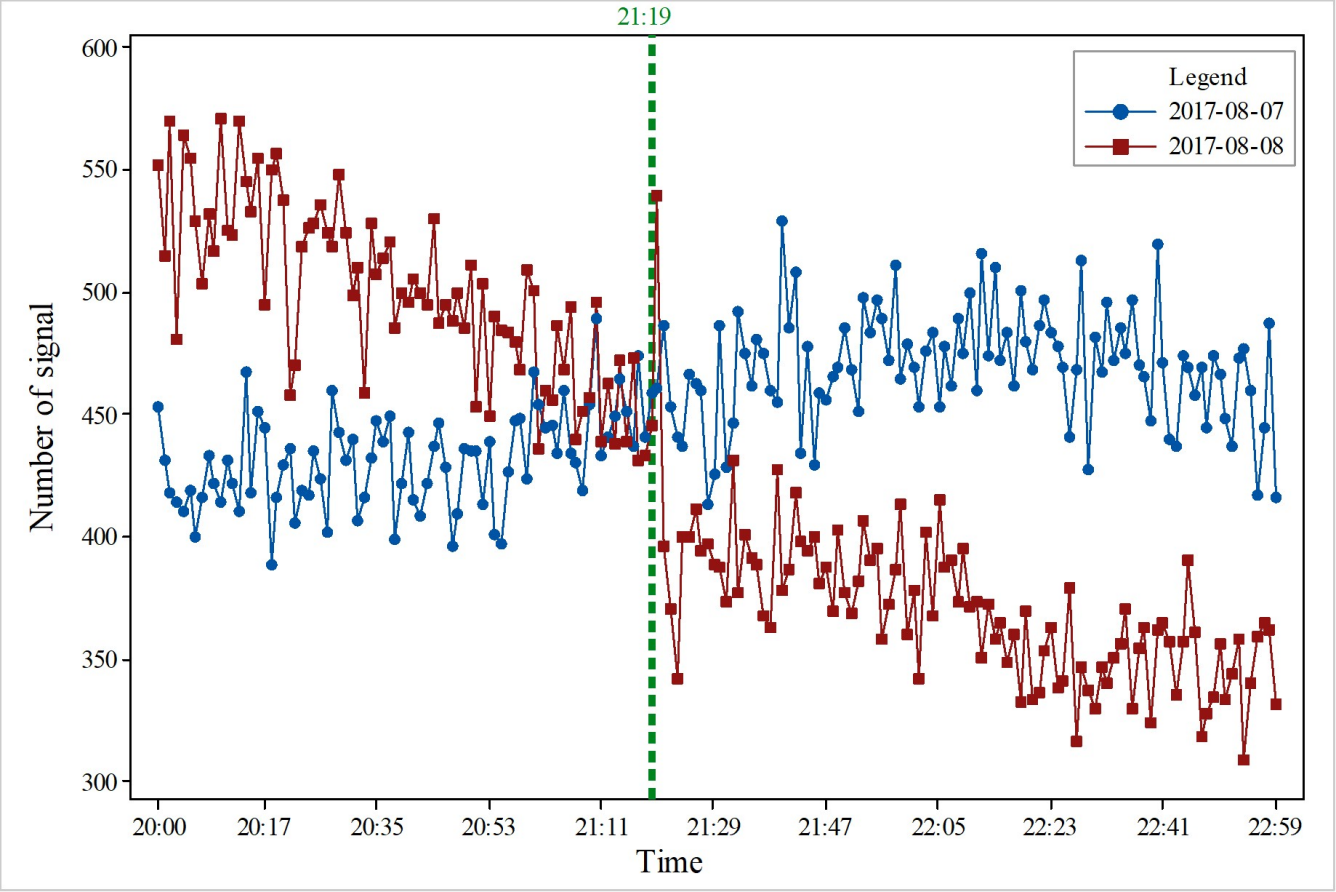
Changes in cell phone position signal data at different times in the study area.

To obtain accurate changes in the number of signals, we conducted a comparative analysis on an hourly basis. We selected data from one hour before the earthquake, one hour after the earthquake, and the time of the earthquake. As Fig 3 shows, the results indicate that before the earthquake, the total amount of data was maintained at more than 500 on August 8, and the total amount of data was maintained at less than 500 on August 7; the change in quantity increased or decreased, and there was no obvious trend to the change. The trend of increase and decrease was relatively stable. In addition, it did not change much. In Fig 4, the total amount of data was maintained at more than 400 on August 7, and there was no obvious trend to the change. Nevertheless, on August 8, the number decreased after the earthquake, and the data volume changed dramatically an hour after the earthquake. The total amount of data was approximately 350, and the reduction in the signal number was great. The main reason is that people moved to the outside area. In Fig 5, 1 hour after the earthquake, the number of mobile phone signal changes fluctuated; the total signal was relatively stable and between 400–500 on August 7, but the total signal was relatively stable and between 350–400 on August 8. At 22:00 on August 7, the number of mobile phone signals did not change significantly from the previous two hours. The number of mobile phone signals at 22:00 on August 8 was lower than the amount of signals on August 7, indicating that the occurrence of the earthquake had an impact on the number of mobile phone signals. The mobile phone signal data at 1 hour after the earthquake did not show a relatively large increase or decrease, which also suggests that at 1 hour after the earthquake, the responses of people were stable and that the various emergency measures of earthquake were relatively effective. Fig 6 also clearly illustrates that the number of mobile phone signals on August 7 was between 400 and 600. There was no obvious fluctuation and change with time, but the number of signals on August 8 showed a large change at the time of the earthquake, and the number of mobile phone signals after the earthquake was significantly lower than the numbers at times before the earthquake or the number at the same time before the earthquake.

**Fig 7:**
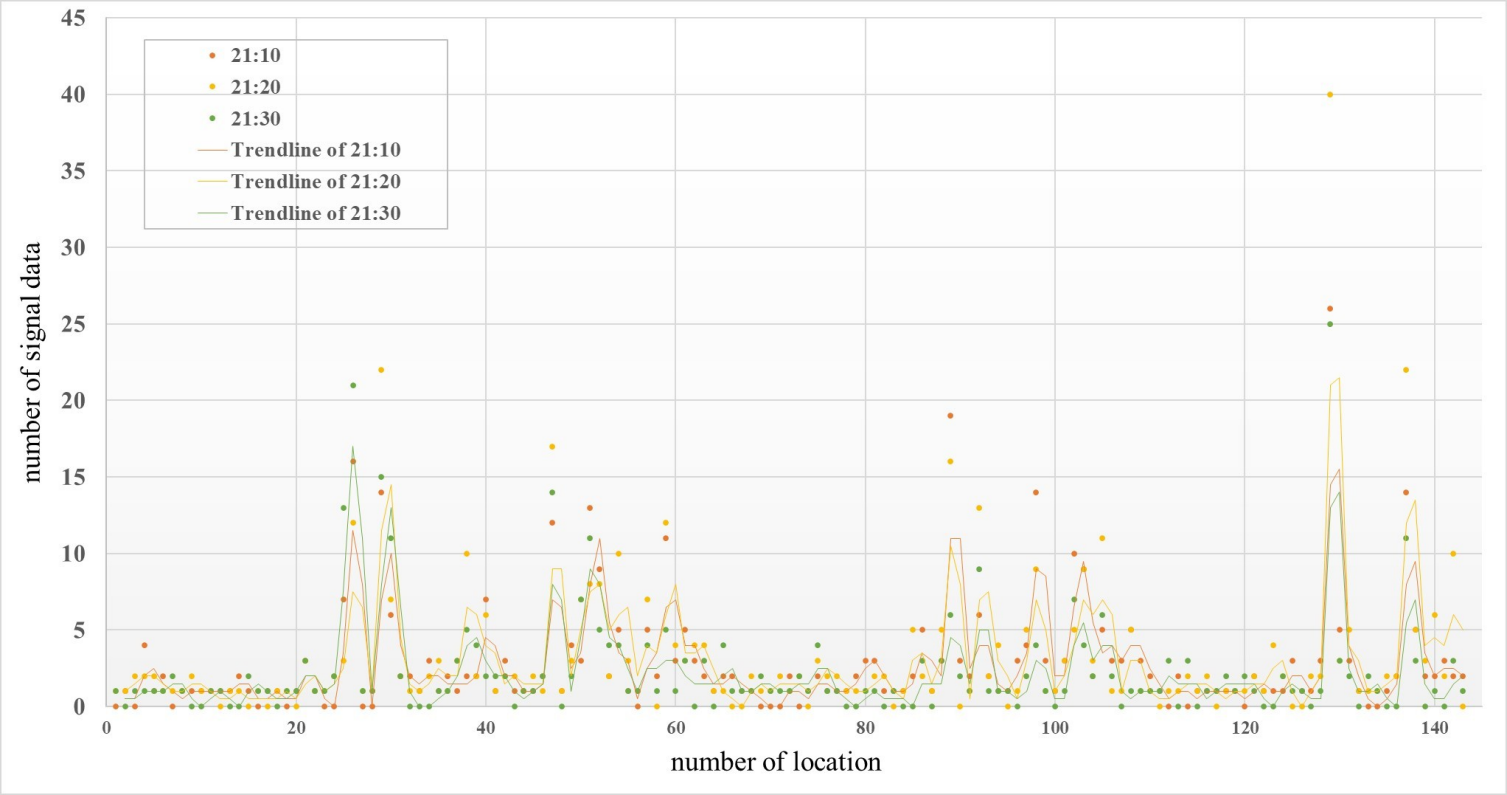
Mobile phone location signal data at different times with respect to the earthquake.

As shown in Fig 7, the number of signals in different locations was more variable, with some regularity in specific geographic locations, and the number of signals in 3–4 areas was relatively higher. Through judging location, these areas were mainly concentrated in Jiuzhaigou County, such as the county area. The other data volumes were relatively small, mainly in rural areas. From the data measured at different times in the same area, the amounts of data change before and after the earthquake are larger and more obvious in the Jiuzhaigou County area, which contained the largest amount of data at the time of the earthquake.

**Fig 8:**
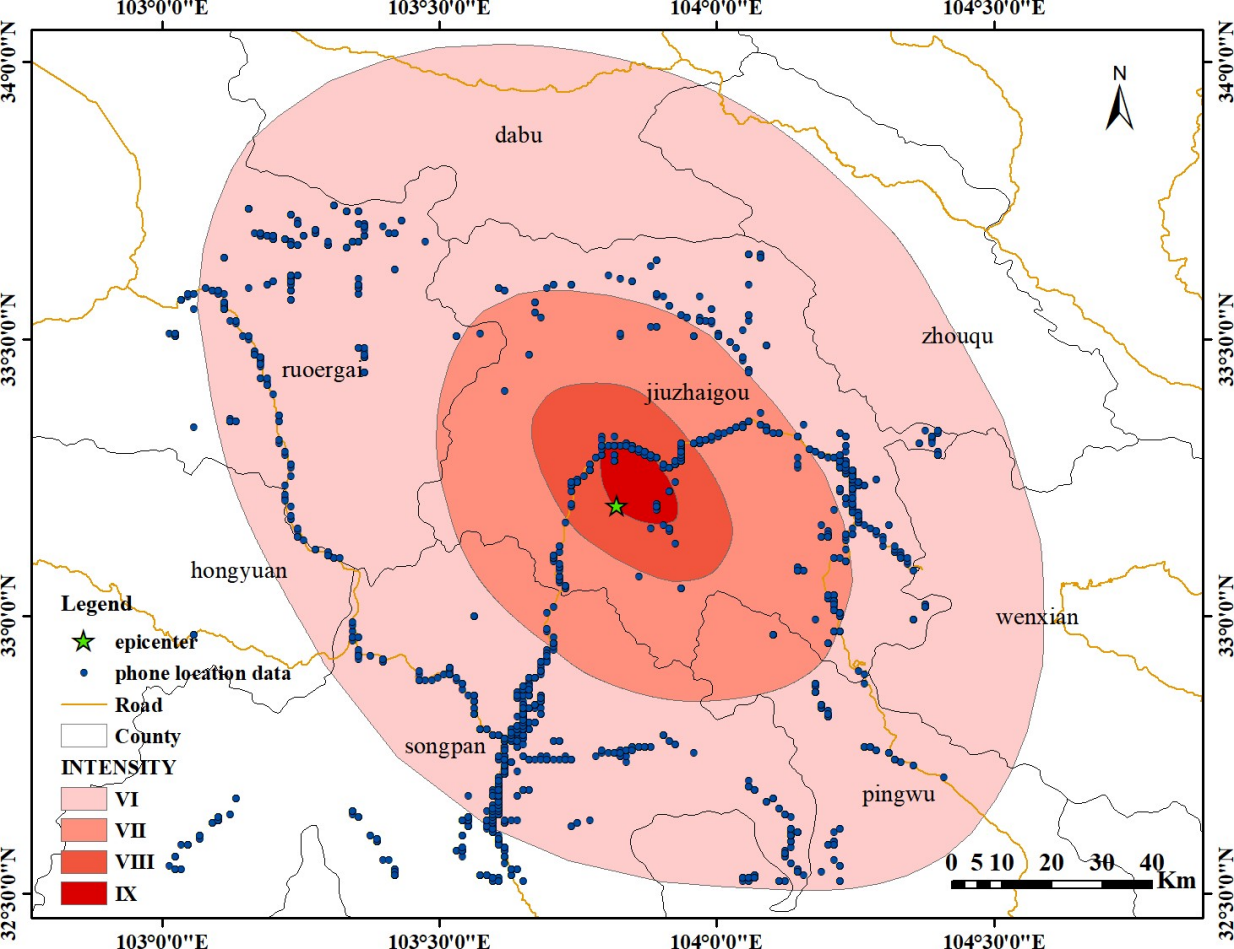
Mobile phone signal location distribution in the study area.

The overall number of mobile phone locations in the study area is shown in Fig 8, which shows the location of the mobile phone data collected around the epicenter at the time of the earthquake at 21:19. The distribution of the signals has certain regularities. Signals are mainly distributed along the road near the epicenter. This situation is in line with the characteristics of the Jiuzhaigou region. Jiuzhaigou County is located in a mountainous area, and the distribution areas of buildings are mainly concentrated on both sides of the valleys. According to the seismic intensity map of Jiuzhaigou released by the China Earthquake Administration, the amount of signal data is also mainly distributed within the intensity VI region, most of which are concentrated within intensity VI circles, including Jiuzhaigou County, Ruoergai County, Songpan County, and Pingwu County. The distribution is relatively decentralized and mainly distributed along the roads, and the distribution of the number of signals in the intensity VII, and VIII zones is mainly along the roads. We extracted and displayed the signal data in chronological order. The results showed that the signal data distribution range was relatively fixed, mainly along the roads, and relatively distributed in the northern rural areas, whether before, during or after the earthquake stage.

**Fig 9:**
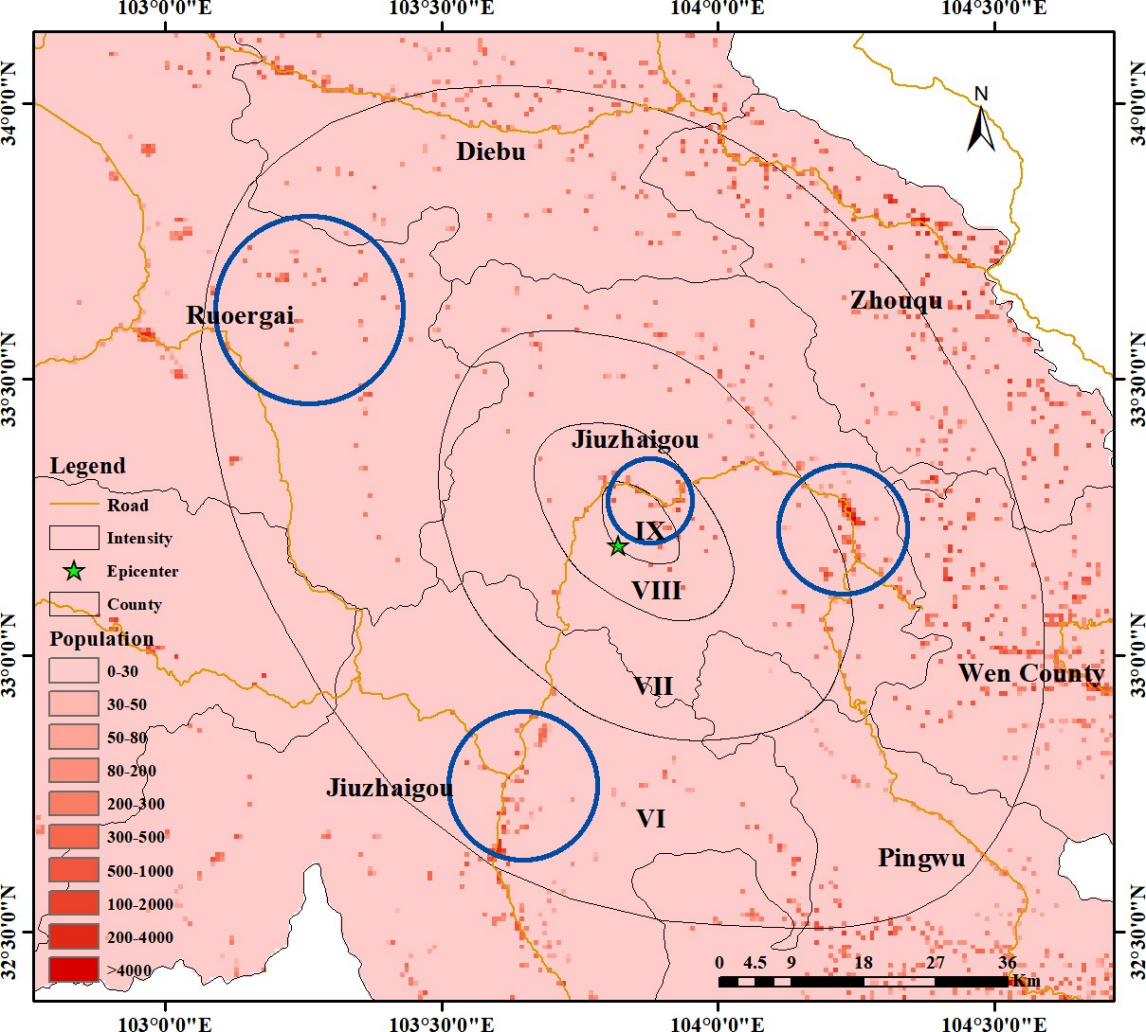
Population distribution in the study area.

As shown in Fig 9, the population distribution of the study area is mainly concentrated in the county area. Among these areas, the distribution is mainly along the roads, the population density is relatively large, and the personnel are relatively dense. Comparing Fig 9 with Fig 8, the signal distribution of mobile phones can be found. The area shows a similar pattern in the distribution position. Further, the population distribution in the study area within the seismic intensity range is mainly concentrated in the area indicated by the blue circle around Jiuzhaigou County in Fig 9, similar to the cell phone signal distribution area. Therefore, the mobile phone signal data can reflect the distribution of the population, and the changes in the mobile phone signals can reflect the actual changes in personnel.

**Fig 10:**
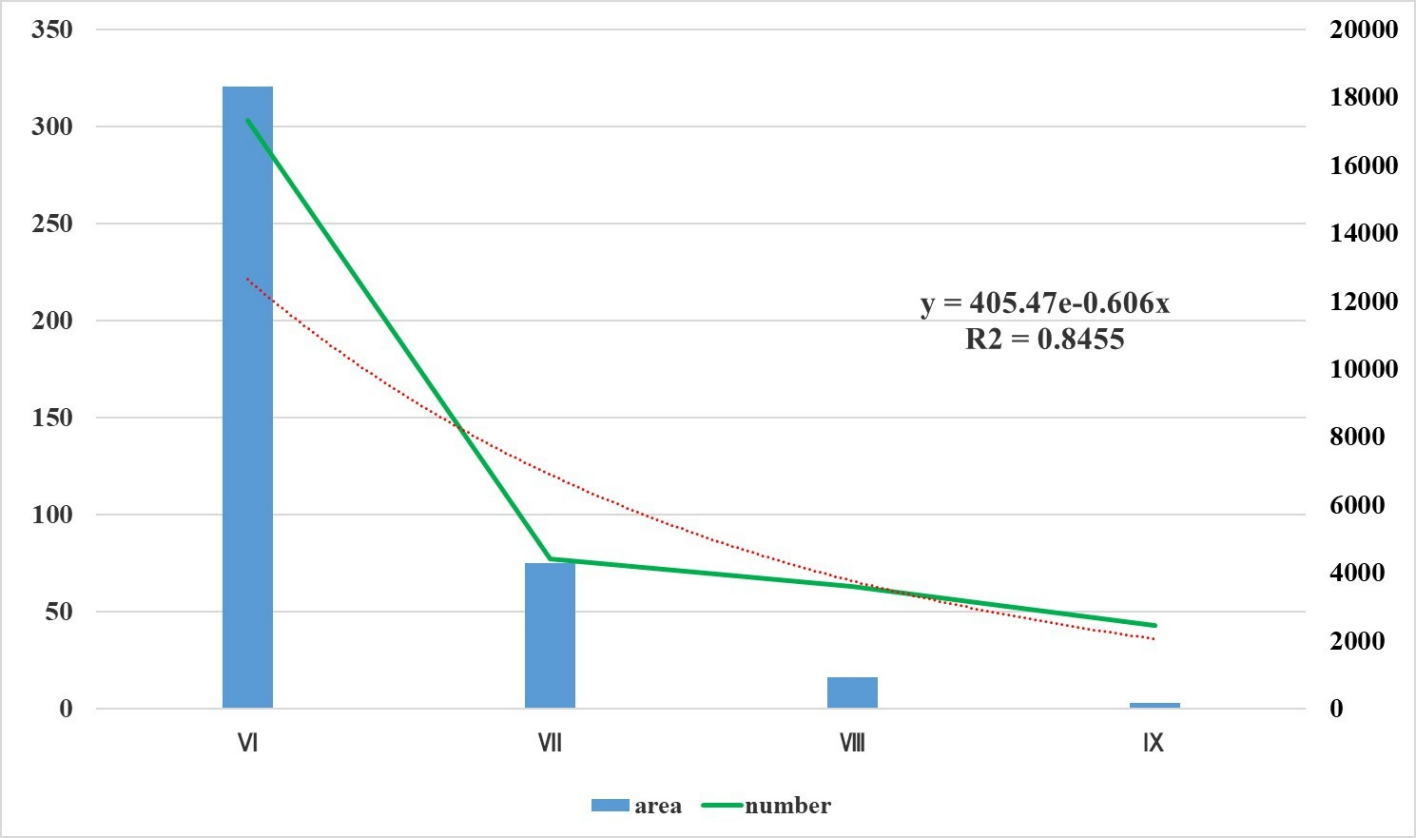
Area and number of signals in each intensity area.

In Fig 10, we performed a fitting analysis of the mobile phone location signal data in the study area according to the intensity range of the Jiuzhaigou earthquake. From the intensity VI region to the intensity IX region, the area gradually decreases, and at the same time, mobile phone signal data in this region show a decreasing trend. Therefore, in order to illustrate the regularity of cell phone signal distribution, we conducted a correlation analysis of the distribution of signal data in each study area and built the relationship between the intensity region and the cell phone signals:

$$I=405.76e^{-0.606N}$$ (2)

The goodness of fit is represented by R^2^, and the value ranges between 0 and 1. If R^2^ is closer to 1, the goodness of fit is higher, whereas if R^2^ is closer to 0, the goodness of fit is lower. In this paper, the R^2^ is 0.8455, which indicates that fit analysis is good.

### The change rate in the overlap area data

We obtained the rates of change for two phases in the same location of the study area: phase a is the change rate in data from before the earthquake to the time of the earthquake, and phase b is from the time of the earthquake to the time after the earthquake.

**Fig 11:**
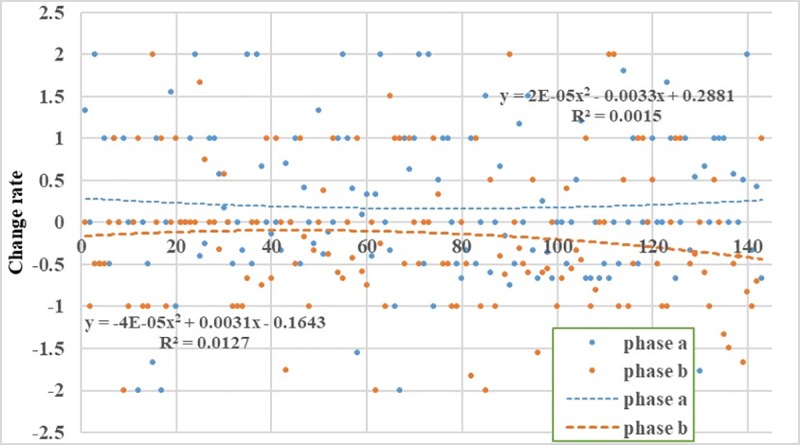
Change rates of overlap data in phase a and phase b.

Fig 11 shows that the change rate in phase a is relatively stable and relatively small. The rate of change ranges from -2 to 2, with the major distribution ranging from -1 to 1, and the size distribution of the change rate is relatively uniform and random. The change rates in the two phases are linearly fitted. The values of R^2^ is small, and the correlations are poor. From this, we can also find that the movement and change of people after the earthquake have irregular. For the change rate of data in phase a, nearly one-third of the number of signal change rates is 0, indicating that within this 10 minutes, the number of signals in these locations did not increase or decrease, which means that the people in this area did not move. Phase a is mainly distributed in the range from 0 to 2; that is, the change in the number of signals in the same area shows an increasing trend. The reason for this pattern may be that the main group of overlapping areas is mainly concentrated in the counties of Jiuzhaigou and Songpan. Because the time is 21:00 in the evening, most of the local residents have entered rest time, but after the earthquake, they use the phone to contact others or release information, which may be the main reason for the increase in the number of signals. In contrast, the change rate of data in phase b is mainly concentrated in the range from -2 to 0; that is, the change in the number of signals in the same area shows a decreasing trend. One of the main reasons for the decrease in the change rate may be that after the earthquake, residents in the earthquake zone move to other regions or the base stations are damaged.

In the 10 minutes before the earthquake, the change rate of mobile phone signals in the study area was mainly concentrated between -1 and 1, favoring the direction of 1, which indicates that the number of signals showed an increasing trend in the 10 minutes before the earthquake. Additionally, at the time of the earthquake, we find from the distance distribution of signal data that the personnel in the study area showed an increasing trend. Second, in the 10 minutes after the earthquake, the change rate of cell phone signals in the study area was mainly concentrated between -1 and 1, favoring the direction of -1, indicating that the number of signals showed a decreasing trend in the 10 minutes after the earthquake. This result means after the earthquake, personnel in the study area showed a decreasing trend.

**Fig 12:**
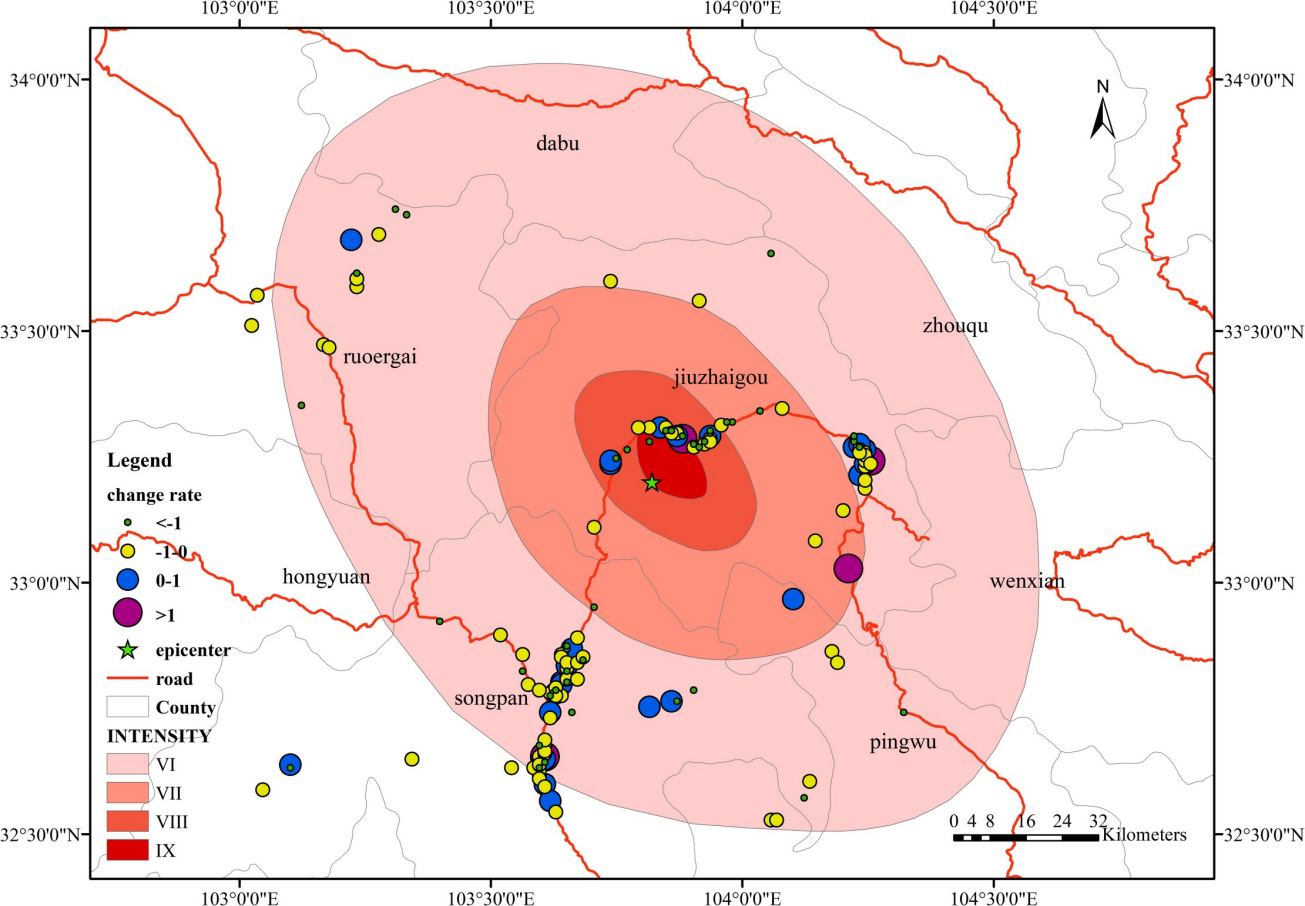
Spatial distribution of the change rate in the overlap area in phase a.

In Fig 12, during phase a, in terms of the spatial distribution of change rates, the areas where the change rate is below 0 are mainly distributed within the intensity VIII and IX zones; the areas where the change rate is above 0 are distributed in the intensity VII and VI zones, mainly in the intensity VI zone. Songpan County and Jiuzhaigou County are predominant. In the time period from the time before the earthquake to the time of the earthquake, the increase and decrease in the number of signals is related to the distribution of the administrative region, but the rate of change is not obvious, and no significant changes occur.

**Fig 13:**
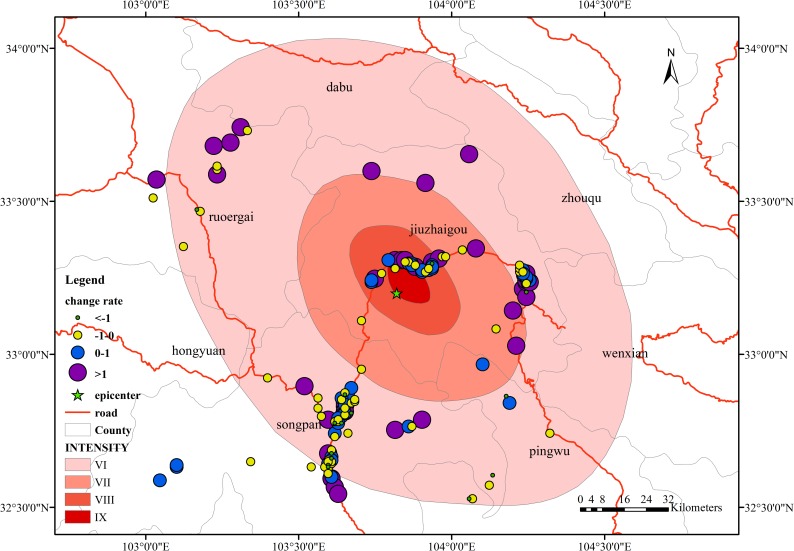
Spatial distribution of the change rate in the overlap area in phase b.

In Fig 13, during phase b, the regions with higher change rates are mainly concentrated in Jiuzhaigou County, including the urban areas in the middle and rural areas in the northwest. These regions are concentrated in the intensity IX and VIII zones, but the change rates in the intensity VII and VI zones, which are farther from the epicenter area, are relatively stable, and they also meet the distribution range of the seismic intensity circle in some respects. The people in high-intensity regions have a strong sense of earthquakes. As a result, the emergency response of personnel is also more immediate, and the change in the number of signals is even more pronounced.

### The change rate in the nonoverlap area data

**Fig 14:**
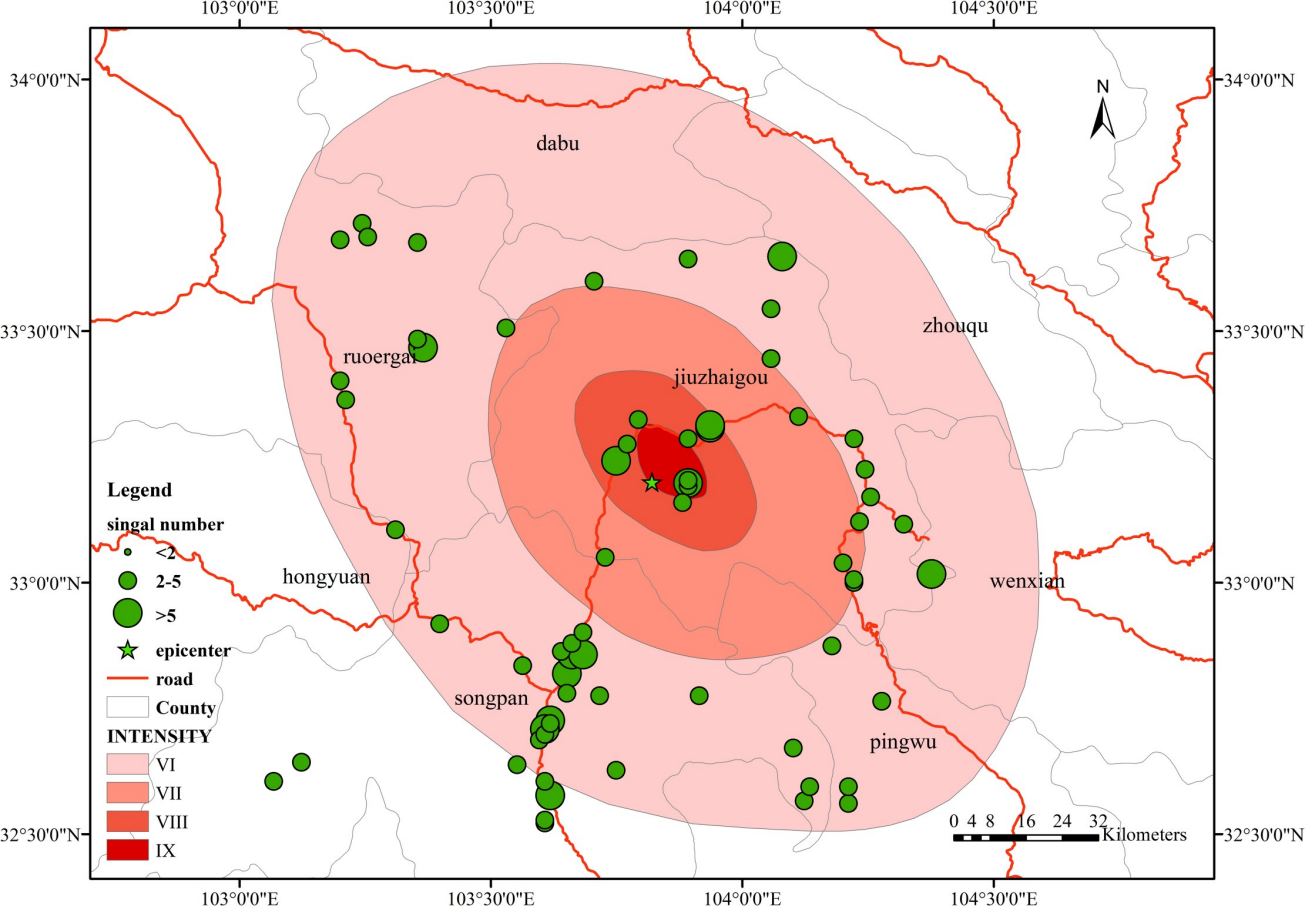
Spatial distribution of change rates in non-overlap areas 10 minutes before the earthquake.

**Fig 15:**
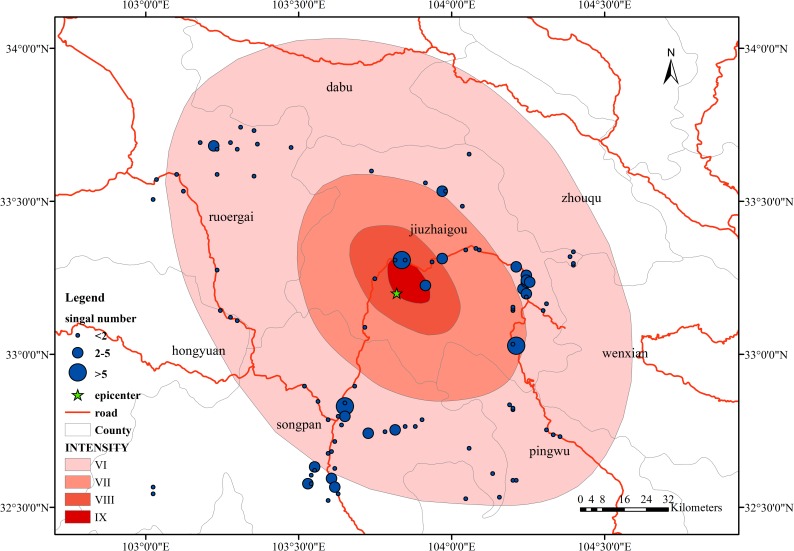
Spatial distribution of change rates in non-overlap areas in the earthquake time.

**Fig 16:**
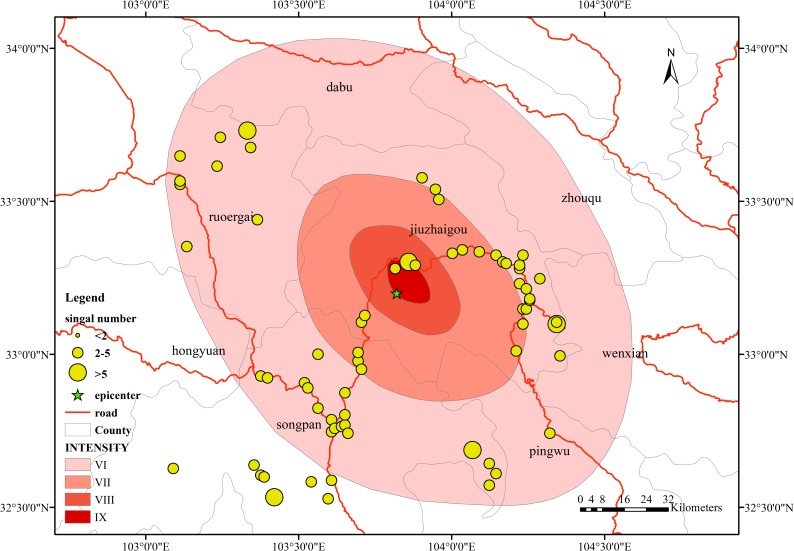
Spatial distribution of change rates in non-overlap areas 10 minutes after the earthquake.

Compared with the data in the overlap areas, there is a certain proportion of nonoverlap areas in the mobile phone signal in the study area. The time period is divided into three phases: 10 minutes before the earthquake (Fig 14), the earthquake time (Fig 15), and 10 minutes after the earthquake (Fig 16). From Figs 14, 15 and 16, in the 10 minutes before the earthquake, the distribution of the data was mainly concentrated near the epicenter, mostly in Jiuzhaigou County and Songpan County, along the roads. The amount of data also has similar regularity; the main source of data is in urban areas. Taking Jiuzhaigou as an example, the amount of data in the county is greater than that in the rural areas in the northwest. The nonoverlap area data at the time of the earthquake does not change significantly compared with the pre-earthquake data, and the data amount and the data change distance do not change dramatically.

We compare the distribution of the data at the time of the earthquake with the data before the earthquake and find a relatively stable movement trend, with certain similarities in concentrated distribution and coincidence with the roads in the county area. The data volume after the earthquake is similar to the data volume before the earthquake, the change is relatively stable, and a large amount of data is concentrated in the county area.

**Fig 17:**
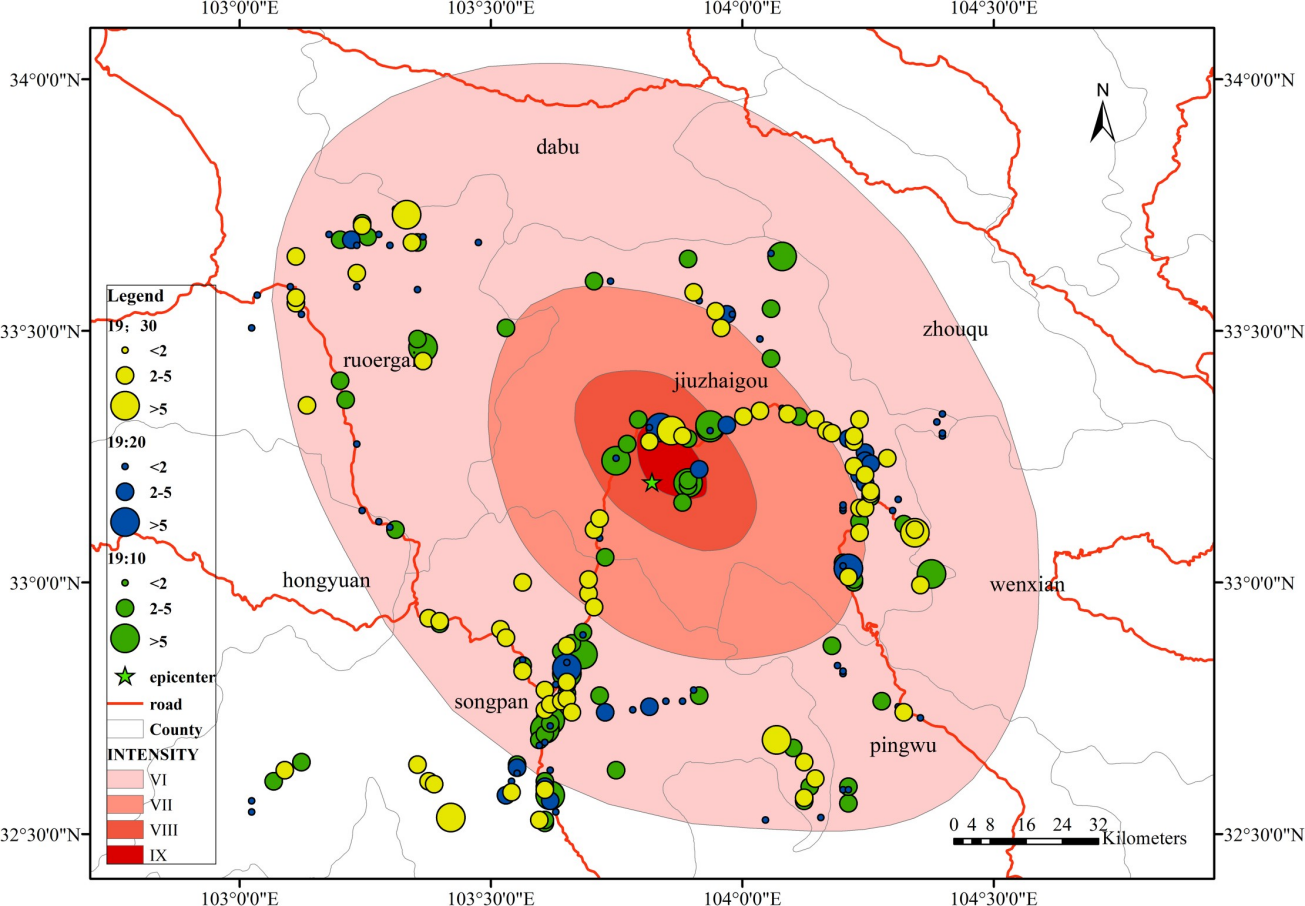
Spatial distribution of change rates in non-overlap areas.

However, from Fig 17, the amount of data in nonoverlap areas after the earthquake is relatively large compared to the previous stage; from the range of distribution, the amount of newly emerged data is affected by the research area, in both the county and rural areas. In contrast, the postearthquake data are relatively few compared to the pre-earthquake data. The distribution in the counties is relatively low. Most of the data are distributed along the roads. When comparing the data from the three periods, the location areas where the data volume appears or increases are certain trends, mainly along the roads, moving and increasing away from the epicenter area. From the data volume point of view, after the earthquake, the data volume of Jiuzhaigou County also shows an increasing trend; however, the increases in the number and density of the Jiuzhaigou County are much smaller than those far from the epicenter. The movement trend of the people after the earthquake is mainly along the roads away from the epicenter.

### Data volume in relation to epicenter distance

Taking the epicenter as the center, we selected buffer zones at distances of 15 km, 20 km, 40 km, and 80 km from the epicenter, and we analyzed the change rate in signal data in different buffer zones. The change in signal data is related to the change in time and the increase in distance.

**Fig 18:**
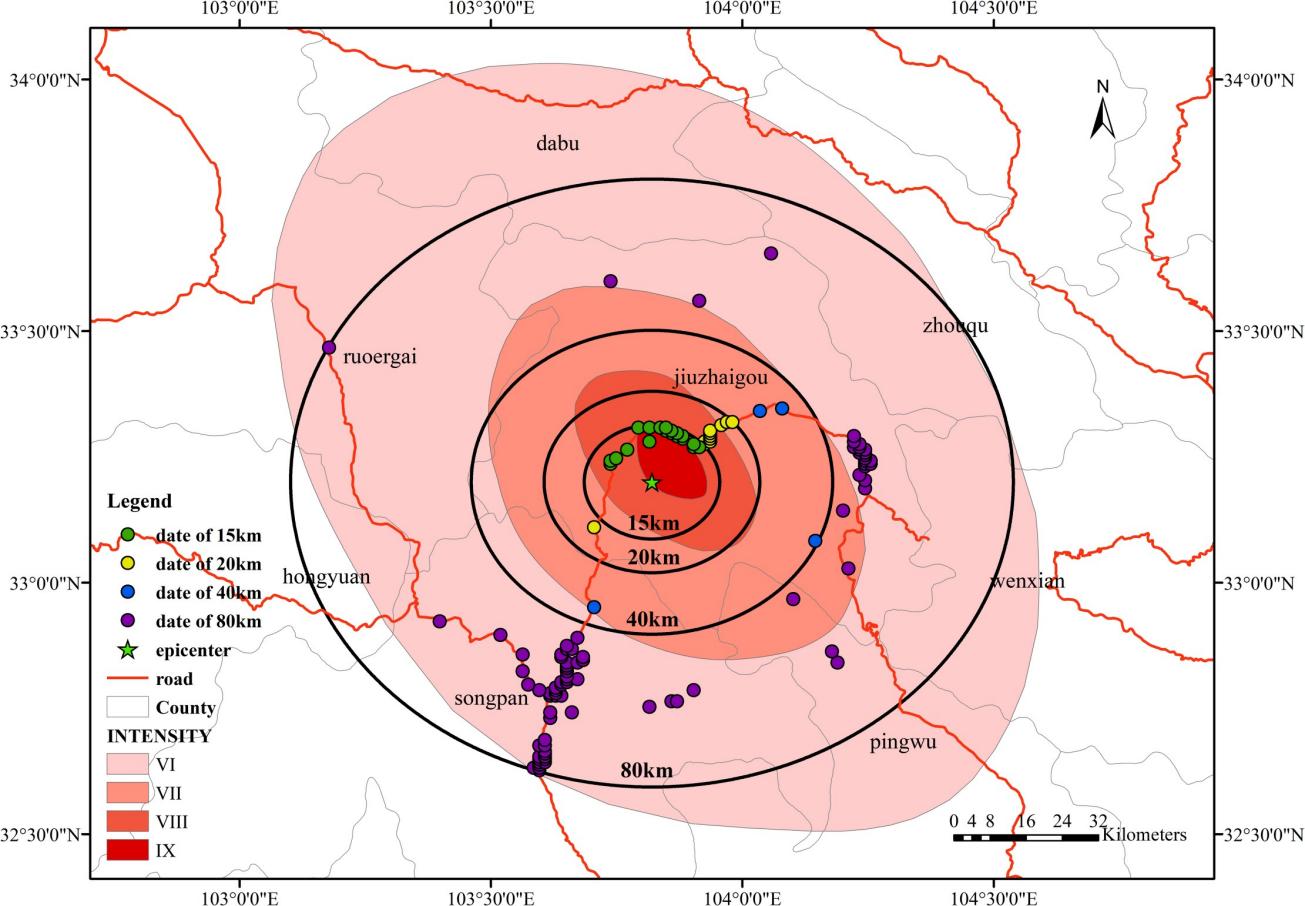
Distribution of data at different distances from the epicenter.

From Fig 18, the important location of mobile phone signal distribution in the study area is within 20 km of the epicentral region; the other region is within the range of 40–80 km from the epicenter, and the dispersion of location distribution is relatively large. An important reason may be that the research area is located in a mountainous area. The topography of Jiuzhaigou County causes the residential areas to be distributed along both sides of the valley roads. The distribution of the residential areas is relatively scattered and distant. With the change in distance, the change rate in the signals within the study area also shows a certain regularity. There are two main areas: one area is within the range of 20 km from the epicentral area. When compared with the seismic intensity map of Jiuzhaigou, the distribution of signal data is mainly for the zone with an intensity of IX. The distribution of signal data within 20–40 km is relatively small, which is related to the geographical area of Jiuzhaigou County. Most of this zone consists of high mountains and has no population distribution. Within the range of 40–80 km, another signal data distribution area in the study area is far from the epicentral area. The change rate of the signal is relatively stable.

**Fig 19:**
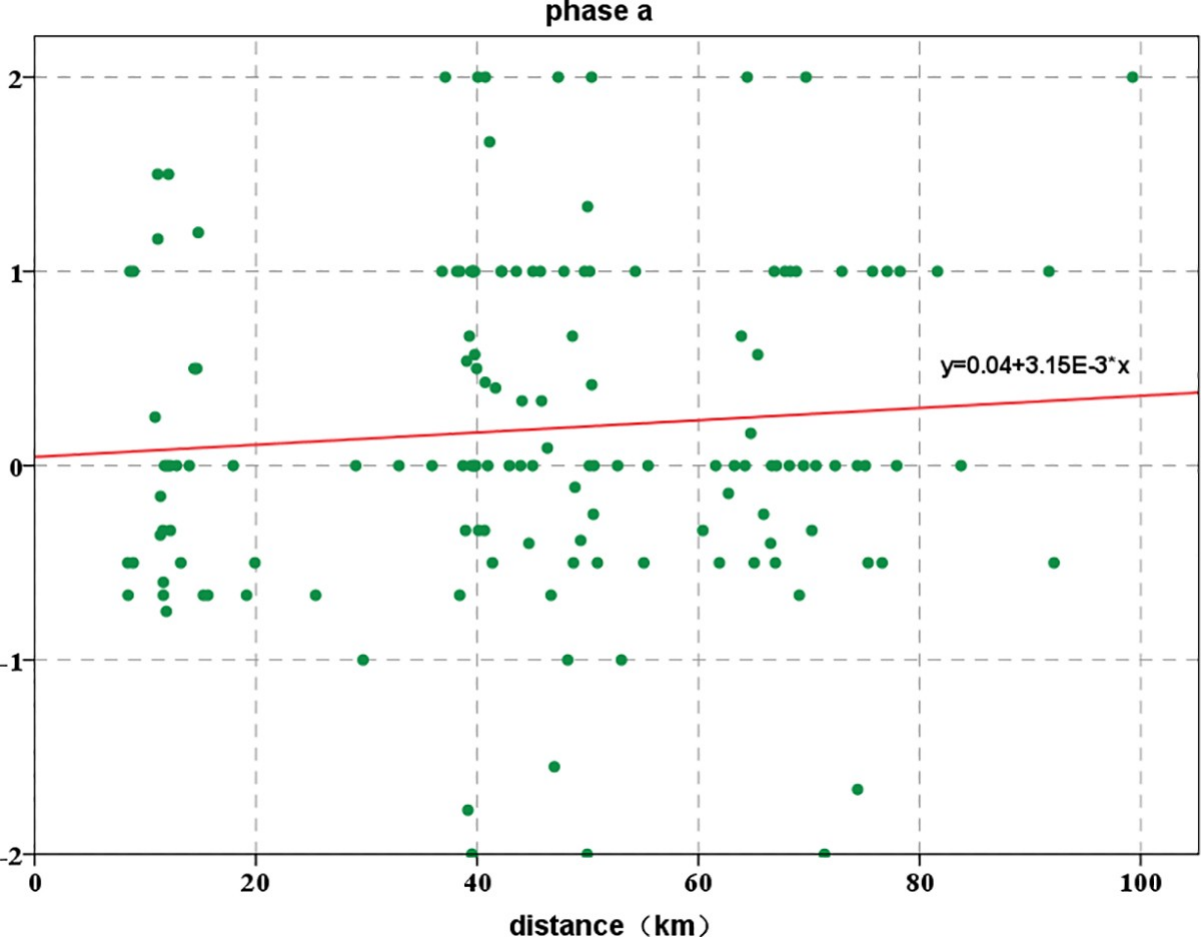
Distribution of data at different distances from the epicenter in phase a.

**Fig 20:**
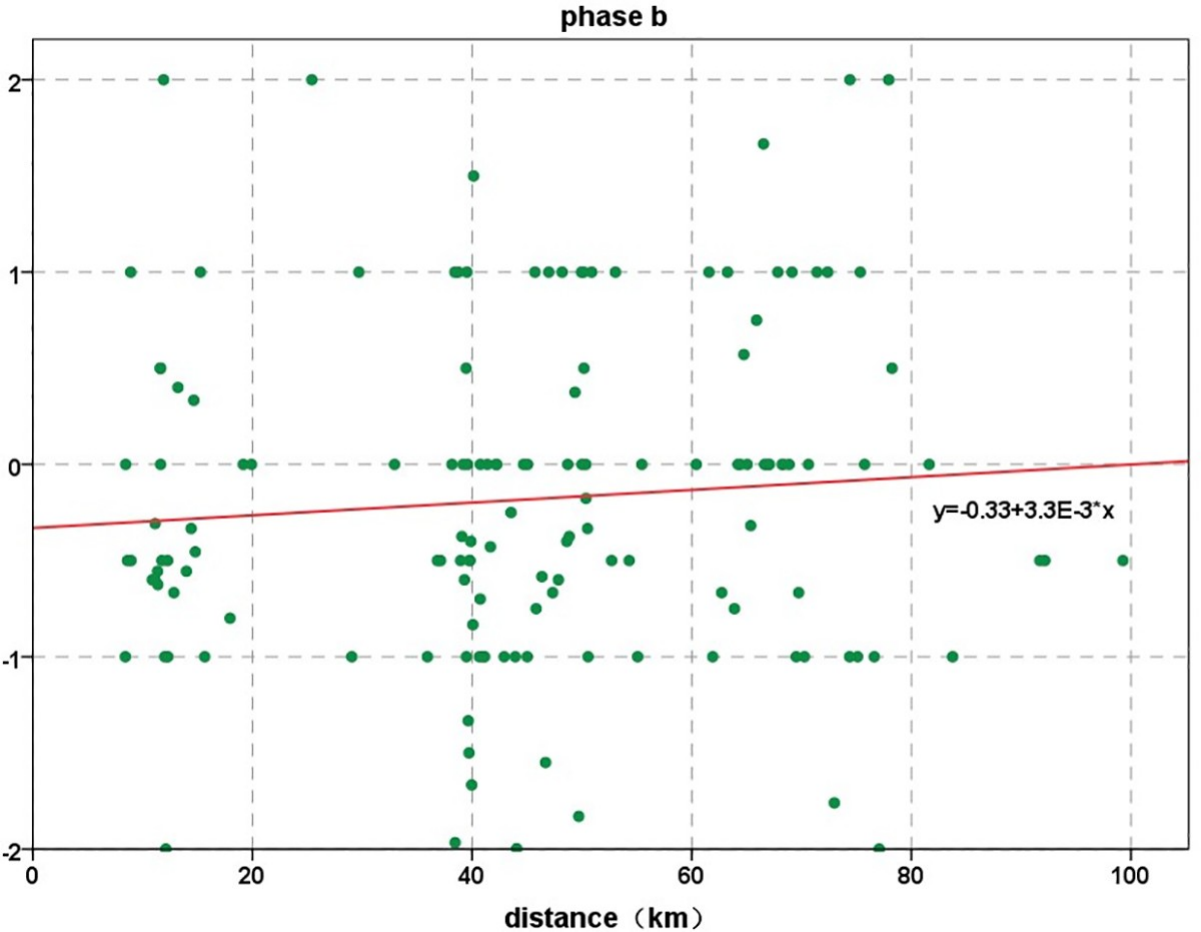
Distribution of data at different distances from the epicenter in phase b.

In Fig 19, the pattern is similar to that of the pre-earthquake data; the distance distribution of the signal data after an earthquake falls mainly in two ranges: one is within the range of 0–20 km from the epicenter, and the other is within the range of 40–80 km. In phase a, the mobile location signal data are mainly distributed in the regions of 0–20 km and 40–80 km. Among them, in the 40–80 km area, the signal number distribution is mainly concentrated in the area near 60 km. This region is mainly Jiuzhaigou County and Songpan County; as in phase a, as show in Fig 20, phase b can reflect a certain regularity to the distribution of the signal in the study area. We can obtain the actual characteristics of the population distribution of the study area by the distribution of the signal data.

## Discussion

In this paper, we present preliminary results to characterize the social response to an earthquake. We used the Jiuzhaigou earthquake as an example; we obtained mobile phone location signal data 2 hours before the earthquake and 8 hours after the earthquake in Jiuzhaigou and determined the behavior patterns of the people after the earthquake based on the distribution of and changes in the mobile phone location signal data. In contrast, we also collected data for the same period on the day before the earthquake. The distribution of personnel and the regular pattern of activities provide data support and a theoretical basis for earthquake emergency decision making. Our results show that the total amount of signal data in the study area can be divided into several stages:

1. In the first stage, the pre-earthquake period, the total amount of data is relatively large. Moreover, the change in the total amount of signals is generally more stable, and the amount of data change is small. The number of signals per minute was concentrated in the range of 200–400. The number of mobile phone signals currently obtained is only the number of signals that can be collected. It cannot represent the actual number of people in the local area. The actual population may be higher than the number of mobile phone position signals we have obtained. However, the obtained mobile phone location signal data can reflect the actual real-time personnel response.
2. In the second phase, within 1 minute after the earthquake, the data volume fluctuates markedly and rapidly decreases. The total signal reduction ratio is approximately 26.7%. The main reason is that after the earthquake, the evacuation of people in the earthquake area to outside of the earthquake area caused rapid signal data in the earthquake area. A second possible reason is that due to the decommissioning of the communication base stations, the amount of signal collection is small. According to the Sichuan Communications Administration's announcement, the base stations in the study area were damaged. From Sichuan Telecom, Jiuzhaigou has 78 base stations, and 31 base stations were affected; from Sichuan Unicom, Jiuzhaigou has 97 base stations, and 65 base stations were affected, and local transmission from Songjing to Hongyangou was interrupted. From Sichuan Mobile, Jiuzhaigou has 360 base stations, 138 base stations were damaged, and the cable from Gonggangling to the Convention and Exhibition Center was interrupted. From Sichuan Iron Tower, Jiuzhaigou has 410 base stations, 21 base stations were affected, and the overall rate of decommissioning was approximately 27% (http://www.scca.gov.cn/2/2/2017-08-09/2834.html).
3. The other phase of change is the early morning on August 9, the number of signals has increased from 100 to nearly 500, one reason is the large-scale evacuation of tourists and residents, and another reason is the arrival of rest time, both of which resulted in the rapid reduction in the number of signals after the earthquake. Moreover, with various rescue teams arriving in succession, the number of signals returns to normal levels. Overall, the change in the number of signals is consistent with the social response after the earthquake. After the earthquake, due to the decommissioning of the base stations and the evacuation of personnel, the number of signals decreases. At the same time, with the arrival of various rescue teams, the number of signals increases.
4. 10 minutes after the earthquake, the changes in signal volume are more frequent, but the total amount is relatively low compared to the intensity. We compare and analyze the mobile phone location signal data of three phases, including the 10 minutes before the earthquake, the seismic moment and 10 minutes after the quake. If the amount of data before the earthquake is smaller, the amount of data after the earthquake is minimized. Thus, there is a tendency to increase and then decrease. The reason for this phenomenon may be that there are more earthquake responders when the earthquake occurs, especially in the earthquake area. As time passes, the number of users gradually becomes stable or moves outside the seismic zone, causing the signals to decrease after the first increase.
5. The distribution of mobile phone location signal data is also in line with the objectivity of the earthquake. The epicenter of the earthquake is located in the Jiuzhaigou scenic spot. The time was 9 o'clock in the evening, and the population is relatively small. Therefore, relatively few data can be collected. At the same time, due to the highest intensity of the earthquake reaching IX, some base stations cease to operate, which is one of the reasons for the decrease in the number of signals. From the perspective of the phone location data, the real-time signal number can reflect the personnel distribution in the earthquake area, especially the personnel distribution within the scope of the seismic influence area. We are able to intuitively locate the concentrated distribution area of personnel, and we can in a timely manner find the area where rescue may be needed. At the same time, given the signal quantity and distribution location changes minute by minute, these data can reflect personnel movement trends. The mobile phone location signal data can reflect the real-time situation of the earthquake area directly and can provide the basis data for emergency decisions.
6. The total amount of signal data in the study area increased rapidly approximately 3 hours after the earthquake, and the main reason for this phenomenon may be the arrival of various disaster relief teams in the earthquake area, causing a sudden increase in the number of signals. We can quickly determine the distribution characteristics of people after an earthquake based on the phone location data. We can determine the possible aggregation areas of people based on the distribution of the data. From the point of view of the distribution of signal data, these data are mainly distributed along roads. The distribution of people is mainly in counties, mostly in Jiuzhaigou County, Songpan County, and Ruoergai County. Since the personnel in the study area are mainly distributed along the roads, the smoothness of the roads after an earthquake is the key to emergency rescue.
7. Through the change rate of the phone location data, we can obtain the law and trend of change. Through the change in the amount of data in the same area before and after the earthquake, we can determine the change of personnel in the area. An area that decreases may be the disaster point. Moreover, the increased area may be a relatively empty shelter, etc. For nonoverlapping regions, the data in the region change every minute. We can determine the flow direction of the postearthquake personnel based on the changes in the time series, which can provide a basis for the rapid planning of the rescue evacuation routes after the earthquake. By studying the changes in the regional data before and after the earthquake, we obtain the change rate of the data in the study area. For the change rate of overlap areas, we find that the data changes in the same area after the earthquake show a decreasing trend. The areas with large change rates after the earthquake are mainly distributed in the county town of Jiuzhaigou, and the rate of change ranges from 0 to -1. Through comparison with the intensity map of the study area, we find that this area is in line with the intensity IX area of the earthquake. Therefore, considering the amount of data, the rate of change can be used as a reference for quickly depicting seismic intensity after an earthquake. For nonoverlap area data, the changes in data volume in the three practical phases of the earthquake reflect not only the changes in data but also the movement of people. We find that before the earthquake, most of the people were concentrated in the area of the county. After the earthquake, the scope of signal data was mainly distributed outside the county. People's movement trends are mainly distributed in the direction away from the epicenter and along the roads.
8. For overlap area, the change rate of mobile phone signals in the study area was mainly concentrated between -1 and 1, favoring the direction of 1, which indicates that the number of signals showed an increasing trend in the 10 minutes before the earthquake. In the 10 minutes after the earthquake, the change rate of cell phone signals in the study area was mainly concentrated between -1 and 1, favoring the direction of -1, indicating that the number of signals showed a decreasing trend in the 10 minutes after the earthquake. The areas where the change rate is below 0 are mainly distributed within the intensity VIII and IX zones; the areas where the change rate is above 0 are distributed in the intensity VII and VI zones, mainly in the intensity VI zone. Songpan County and Jiuzhaigou County are predominant. In the time period from the time before the earthquake to the time of the earthquake, the increase and decrease in the number of signals is related to the distribution of the administrative region, but the rate of change is not obvious, and no significant changes occur. After the earthquake occurred, the mobile phone signal increased greatly away from the epicenter area, indicating that the flow trend of the personnel after the earthquake is moving away from the epicenter area. For overlap areas, the rate of change in the number of cell phone signals may indicate the distribution of people within the area at different times. The security situation of the area can be determined by the rate of change of different time periods. After the earthquake, people mainly concentrated in urban areas, and the distribution location is relatively concentrated, mainly concentrated in the Jiuzhaigou County and Songpan County.
  Compared with the data in the overlap areas, there is a certain proportion of nonoverlap areas in the mobile phone signal in the study area, in the 10 minutes before the earthquake, the distribution of the data was mainly concentrated near the epicenter, mostly in Jiuzhaigou County and Songpan County, along the roads. The amount of data also has similar regularity; the main source of data is in urban areas. the amount of data in non-overlapping areas after the earthquake is relatively large compared to the previous stage; from the range of distribution, the amount of newly emerged data is affected by the research area, in both the county and rural areas. Most of the data are distributed along the roads. When comparing the data from the three periods, the location areas where the data volume appears or increases are certain trends, mainly along the roads, moving and increasing away from the epicenter area. the data volume of Jiuzhaigou County also shows an increasing trend; however, the increases in the number and density of the Jiuzhaigou County are much smaller than those far from the epicenter. The movement trend of the people after the earthquake is mainly along the roads away from the epicenter. For non-overlapping areas, the rate of change in the number of cell phone signals can more clearly reflect the distribution and flow of people after the earthquake. Especially with the change of time, we can get the accurate and rapid flow trend of people according to the distribution of the change rate of different regions in a fixed time period. The flow direction of the personnel after the earthquake is mainly from the Jiuzhaigou county towns located in the area of intensity of IX and VIII to the Songpan County and other areas of the intensity of VI and VII.
9. The relationship between data volume and the epicenter is relatively obvious. One region is mainly concentrated in the area of intensity IX, mostly in the Jiuzhaigou County area, within 20 km of the epicenter, and the other relatively concentrated area is in the intensity VI region, 80 km from the epicenter, relatively far away. However, the number of signals in the intensity VII and VIII regions of the earthquake is relatively small. One of the main reasons is the influence of topography and landforms in the study area, which is mostly mountainous with few residents.

## Conclusions

This paper analyzes the feasibility of using mobile phone location signal data in earthquake emergency rescue work from several aspects, such as quantity, location, change rate, and epicentral distance. The results show that the mobile phone location signal data can quickly obtain the situation of the personnel distribution and quantity after the earthquake, and we find the change rate, the distance, etc. can determine the approximate range of the earthquake impact field. The change in data volume can well reflect the postearthquake movements of personnel. Through the change rate of data volume, people's flow trend can be obtained. We can obtain the concentrated distribution area of the post-earthquake personnel by the rate of change of the mobile phone signal data in the overlapping area. And it is also possible to judge the flow direction of the personnel after the earthquake due to the rate of change of the signal data of the mobile phone in the non-overlapping region. After the earthquake, people mainly concentrated in urban areas, and the distribution location is relatively concentrated, mainly concentrated in the Jiuzhaigou County and Songpan County. The flow direction of the personnel after the earthquake is mainly from the Jiuzhaigou county towns located in the area of intensity of IX and VIII to the Songpan County and other areas of the intensity of VI and VII.

After the earthquake, the use of mobile phone location signal data can quickly provide a picture of the emergency situation of people in the earthquake area. We can obtain the social response characteristics after the earthquake. Through the data distribution in different time periods, the movement of personnel after the earthquake can be determined. Based on several situations, we can obtain the basic situation of the disaster-stricken areas in times after the earthquake, especially the personnel relevant to the situation, and these data can provide theoretical and scientific support for postearthquake emergency rescue decision making, especially black box emergency rescue work.

We acknowledge that our results are greatly affected by different magnitudes of earthquakes and different regions of earthquakes, such as the impact of magnitude on the distribution and changes of mobile phone location data. The location of the earthquake has an impact, such as the difference between densely populated urban areas and areas with smaller rural populations. Due to the influence of the original data obtained in this paper, we have obtained the mobile phone location data for only the Jiuzhaigou earthquake, but the data for different magnitudes or different regions have not been obtained. For different earthquakes, we have not been able to conduct a comparative study.

Mobile phone location signal data have value in many previous uses, especially the real-time and dynamic characteristics of data that can be used for disaster relief, traffic monitoring, population census, etc. In future studies, we will use this technique in combination with other parts of emergency operations, such as food support and shelters.

## Supporting information

S1 File The phone signal number data of jiuzhaigou earthquake. (XLSX)
